# Ectopic Liver Tissue on the Gallbladder Wall Encountered During Laparoscopic Cholecystectomy

**DOI:** 10.7759/cureus.57088

**Published:** 2024-03-27

**Authors:** Hajime Imamura, Ken Taniguchi, Mampei Yamashita, Tomohiko Adachi, Susumu Eguchi

**Affiliations:** 1 Department of Surgery, Nagasaki University Graduate School of Biomedical Sciences, Nagasaki, JPN; 2 Department of Surgery, Nagasaki Harbor Medical Center, Nagasaki, JPN

**Keywords:** accessory liver, liver tissue, cholecystectomy, gallbladder, ectopic liver

## Abstract

Ectopic liver tissue is a rare developmental anomaly that is not directly connected to the liver. We encountered ectopic liver tissue on the surface of the gallbladder wall during laparoscopic cholecystectomy. It has vasculature arising from the liver parenchyma and is classified according to its branching pattern. Ectopic liver tissue has been reported to occur in a variety of locations, and when encountered in surgery, it is clinically important to identify ectopic liver tissue with vascular supply to prevent unexpected bleeding. Ectopic liver tissue should be resected and examined histologically for the potential for malignancy when detected during surgical intervention.

## Introduction

Ectopic liver tissue (ELT) is a rare clinical entity. These abnormalities are classified as accessory liver when the hepatic tissue is attached to the native liver and ELT when the ectopic hepatic tissue is not directly connected to the liver [1]. It is often an incidental finding during surgeries, autopsies, or diagnostic imaging. It is usually asymptomatic and may be found incidentally, but when detected, resection is recommended because of the possibility of hepatocellular carcinoma. Although statistical analysis of the frequency of incidence is difficult, it has been reported that 9 out of 70 cases of ELT reported by 1985 developed hepatocellular carcinoma. From this point of view, it appears that ELTs are prone to hepatocellular carcinoma [2]. We experienced a case of resection of an ELT found in laparoscopic cholecystectomy and report it here with some literature review.

## Case presentation

**Figure 1:**
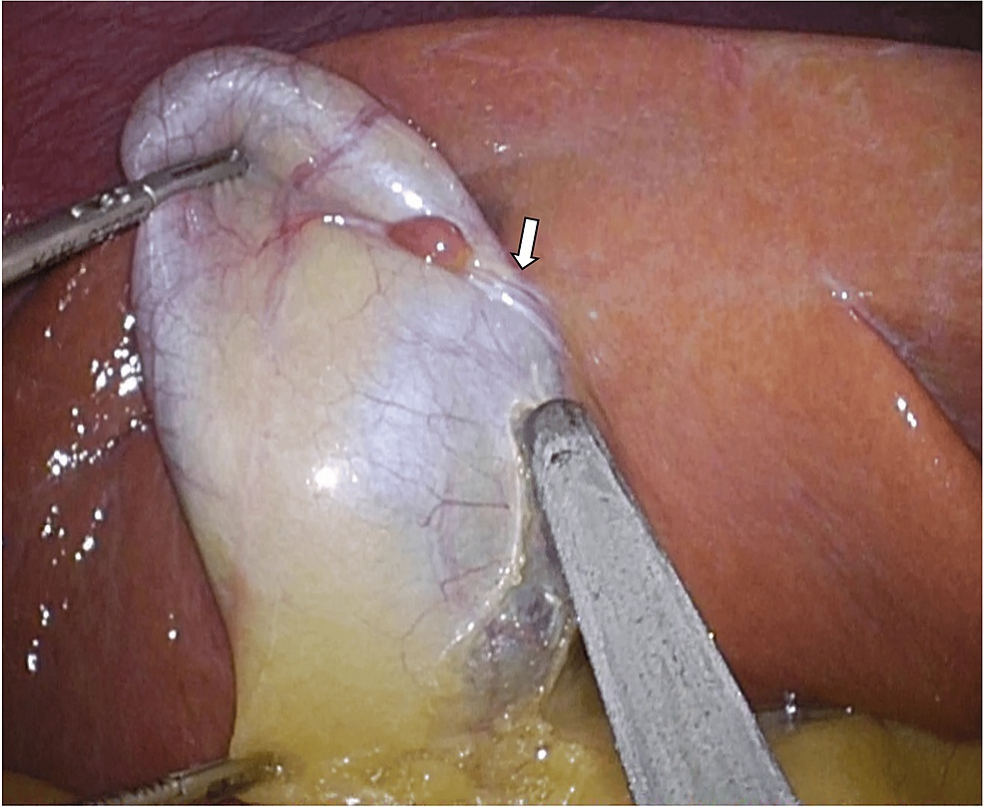
Ectopic liver tissue was detected on the surface of the gallbladder wall. It had a vascular pedicle arising from the liver parenchyma (arrow).

A 40-year-old man presented with gallbladder stones and underwent elective laparoscopic cholecystectomy. During laparoscopic cholecystectomy, ectopic tissue approximately 1 cm in diameter was detected incidentally on the surface of the gallbladder wall, having the same color as the liver. It had a vascular pedicle arising from the liver parenchyma (Figure 1).

The vascular pedicle (Figure 1, arrow) was dissected with coagulation. The ectopic tissue was excised with the gallbladder, and laparoscopic cholecystectomy was finished uneventfully. A histological examination revealed the ectopic liver tissue (ELT) to have no malignant findings. The ELT was not directly connected to the liver. The patient suffered no postoperative complications and was discharged on the third day after surgery.

## Discussion

ELT is a rare developmental anomaly. These abnormalities are classified as accessory liver when the hepatic tissue is attached to the native liver and ELT when the ectopic hepatic tissue is not directly connected to the liver [1]. In general, ELT is asymptomatic and found intraoperatively during surgery performed for other reasons or at an autopsy. ELT was reported in 3 (0.05%) cases at autopsy among 5500 cases, and a review of 1060 laparoscopic procedures found ELT in 3 patients related to the gallbladder (0.23%) [3,4]. In an analysis of 1,060 laparoscopic procedures, only 3 patients were identified with ELTs attached to the gallbladder, with a prevalence of 0.28% [5]. More cases of ELT may be identified during staging laparoscopy with the increase in the prevalence of other types of laparoscopic surgeries (including gastrectomy and colectomy). Ectopic liver can occur in a variety of sites in the body. To date, discoveries have been reported in the heart, umbilicus, lungs, spleen, umbilicus, and vena cava [5-7]. Although ELT is a rare condition, the actual incidence of ELT may be underestimated because of its asymptomatic nature and a lack of awareness among medical staff.

From the embryological viewpoint, there are various theories regarding the mechanism of ectopic liver development. These theories include the development of accessory liver lobes, migration or displacement of liver bud, dorsal budding of hepatic tissue, entrapment of hepatocyte-destined mesenchymal cells, and entrapment of cell nests in the foregut region [6]. However, the detailed mechanism will require further study.

Three different types of vascular supply of ELT attached to the gallbladder were reported by Bal et al. [8] as follows: type 1, artery arising from the cystic artery [9]; type 2, vascular pedicle (with/without its own vein) arising from the liver parenchyma [8]; and type 3, vascular structures embedded in the mesentery lying from the hepatic site to the ELT [10]. The present case corresponds to type 2. It is clinically important to define ELT with a vascular supply during cholecystectomy because traction of the gallbladder may cause the rupture or tearing of the vascular structure from the liver parenchyma.

ELT usually has a normal liver tissue histology (i.e. normal portal structure, regular lobules, and central vein). However, there has been evidence to suggest that ELT is a risk for hepatocarcinogenesis, as are cirrhosis, viral infections, and chemical carcinogens [2]. Biliary drainage may be insufficient and/or the blood supply may be reduced in the ELT. Therefore, ELT is more susceptible to the development of malignancy because it does not have a complete vasculature or ductal system like the normal liver and may be functionally impaired [10].

## Conclusions

In conclusion, ELT may be recognized incidentally during other surgical procedures, and its presence is not well recognized by the medical community. However, since vascularization of the gallbladder surface is observed during cholecystectomy, careful surgery and management are considered necessary to avoid accidental bleeding or other problems. If an ELT is discovered during surgical intervention, it should be resected and the possibility of malignancy should be considered histologically.

Since ELT is an anatomic variant that is asymptomatic and may be unintentionally excised or missed, it is desirable to accumulate cases to spread awareness of it among the medical community and to further understand the anatomic perspective and its pathophysiology.

## References

[1] Collan Y, Hakkiluoto A, Hästbacka J (1978). Ectopic liver. Ann Chir Gynaecol.

[2] Arakawa M, Kimura Y, Sakata K, Kubo Y, Fukushima T, Okuda K (1999). Propensity of ectopic liver to hepatocarcinogenesis: case reports and a review of the literature. Hepatology.

[3] Tejada E, Danielson C (1989). Ectopic or heterotopic liver (choristoma) associated with the gallbladder. Arch Pathol Lab Med.

[4] Watanabe M, Matsura T, Takatori Y (1989). Five cases of ectopic liver and a case of accessory lobe of the liver. Endoscopy.

[5] Akbulut S, Demyati K, Ciftci F (2020). Ectopic liver tissue (choristoma) on the gallbladder: a comprehensive literature review. World J Gastrointest Surg.

[6] Huang W, Xu X, Li T, Zhang H, Chen Y, Li S (2015). Ectopic liver tissue in stomach paries: a case report. Int J Clin Exp Pathol.

[7] Soliman M, Akanbi O, Salem A, Khreis M, Abdel-Latif A (2019). Ectopic liver tissue mistakenly diagnosed as a right atrial myxoma. Cureus.

[8] Bal A, Yilmaz S, Yavas BD (2015). A rare condition: ectopic liver tissue with its unique blood supply encountered during laparoscopic cholecystectomy. Int J Surg Case Rep.

[9] Koh CE, Hunt R (2007). Ectopic liver encountered during laparoscopic cholecystectomy. Asian J Surg.

[10] Martinez CA, de Resende HC Jr, Rodrigues MR, Sato DT, Brunialti CV, Palma RT (2013). Gallbladder-associated ectopic liver: a rare finding during a laparoscopic cholecystectomy. Int J Surg Case Rep.

